# Supplementary material for: Molecular insights into profilin 1-dependent regulation of cellular phosphatidylinositol (4,5)-bisphosphate
Source: J Cell Sci. 2026 Jun 12;139(11):jcs265025. doi: 10.1242/jcs.265025 (PMC13286375; doi:10.1242/jcs.265025)
Supplement: Supplementary information [file joces-139-265025-s1.pdf]

Orenberg et al. Fig S1

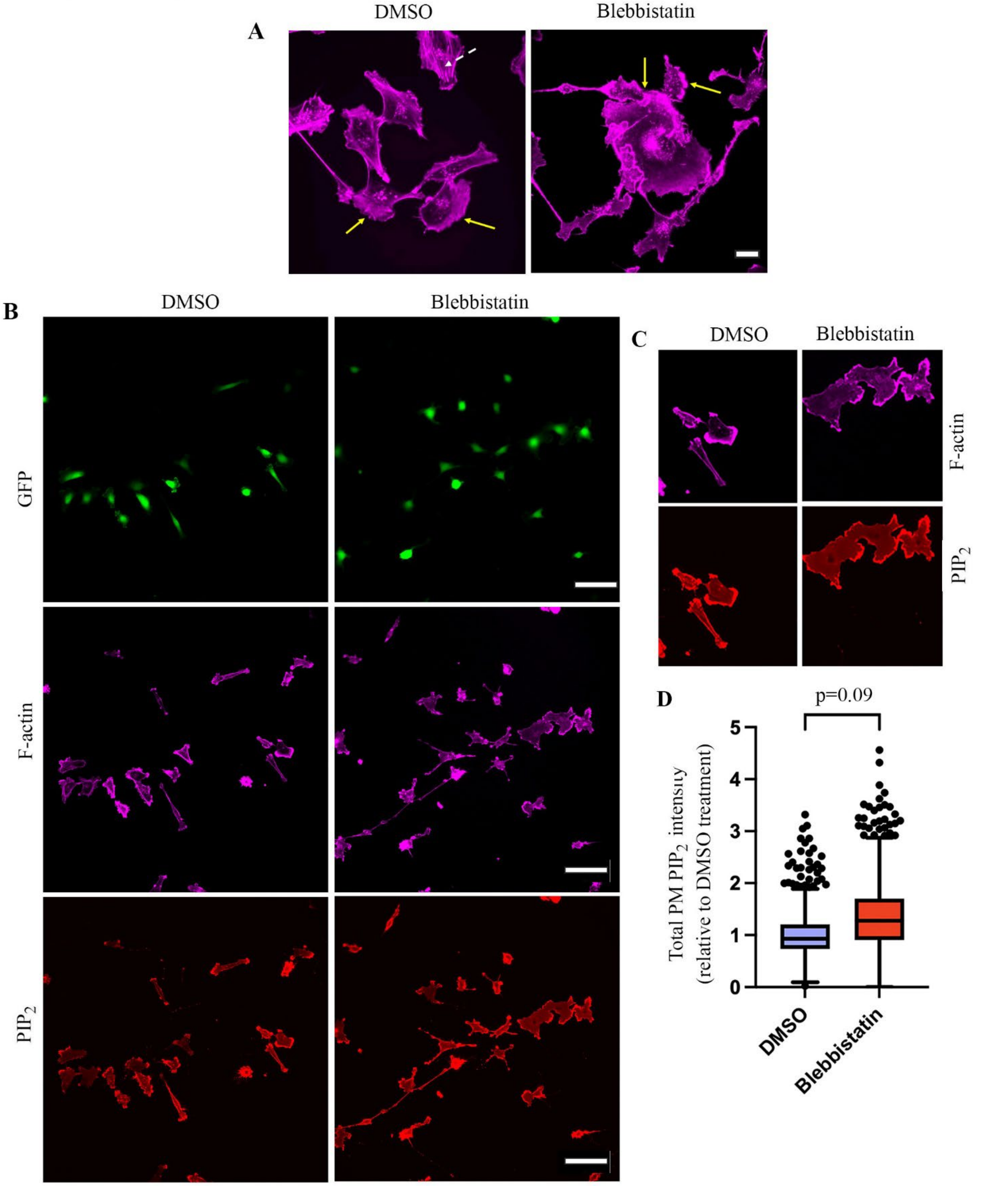

Fig. S1. Effect of blebbistatin treatment on F-actin- and PIP_2 in MDA-231 cells. A) Representative phalloidin staining images (60X magnification) of GFP-expressing subline of MDA-231 cells subjected to 30 min treatment of either blebbistatin or DMSO (scale bar – 20 μm). Arrowheads show actin stress

Journal of Cell Science • Supplementary information

fibers in DMSO-treated cells that were largely eliminated upon blebbistatin treatment. Arrows indicate peripheral F-actin. **B-C)** Representative widefield fluorescence images (20X magnification; scale bar – 40 μm) of phalloidin- and PIP_2-stained GFP-expressing MDA-231 cells (panel B; magnified images of F-actin and PIP_2 staining of selected regions of interest are shown in panel C). **D)** A box and whisker plot summarizing the relative total cell edge PIP_2 staining intensity of the two treatment groups (data summarized from analyses of more than 700 individual cells per treatment group pooled from 3 experiments).

Orenberg et al. Fig S2

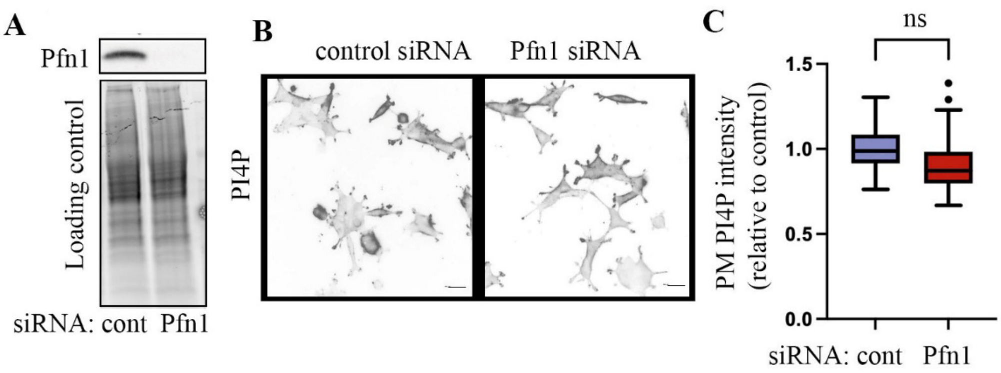

Fig. S2. Pfn1 loss does not impact PI4P content in HEK-293 cells. A) Pfn1 immunoblot (stain-free gel serves as the loading control) of HEK-293 cells transfected with either control or Pfn1-specific siRNAs show efficient knockdown of Pfn1 expression. **B-C)** Representative images of PI4P immunostaining (*panel B;* scale – 50 μm) and quantification (*panel C*) of control vs Pfn1 knockdown cultures (data summarized from 300-400 cells/group pooled from 3 experiments; ns – not significant).

Journal of Cell Science • Supplementary information

Orenberg et al. Fig S3

A

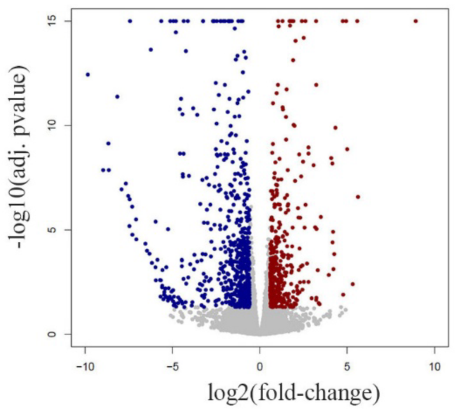

B

DOWNREGULATED			UPREGULATED	
Gene	log2FoldChange		Gene	log2foldchange
FOXP2	-9.85		LOC10192	12.68
NGFR	-8.97		CMKLR1	8.92
CDH11	-8.67		CHRNE	5.62
FAM49A	-8.65		AKR1C1	5.58
CACNA2D	-8.16		KCNJ4	5.32
GABRA3	-7.92		ZBTB32	5.00
VCAN	-7.67		MAGEA11	4.94
TRPM6	-7.54		LINC0122	4.77
ZNF804A	-7.47		AKR1C2	4.75
AMOT	-7.45		C17orf107	4.33
CDK14	-7.43		RSAD2	4.26
FREM2	-7.31		KRT17	4.21
BCHE	-7.30		SORCS3	4.19
SLITRK1	-7.09		CCL5	4.16
TRIML2	-7.07		ELMO1	4.15
TCEAL7	-6.55		NAP1L3	4.09
MAGEA10	-6.46		IFNL1	3.94
COCH	-6.32		CES1P2	3.49
GYPC	-6.24		LINC0064	3.42
LDOC1	-6.18		CH25H	3.37
C3orf70	-5.97		PLXNA4	3.31
PAGE5	-5.97		ITGAX	3.24
RASEF	-5.95		FA2H	3.24
FGF13	-5.86		NCF2	3.23
MARK1	-5.71		LOC10192	3.22
SHROOM4	-5.69		AKR1C3	3.22
LINC00261	-5.67		ZBTB7C	3.17
GPC6	-5.64		C7orf71	3.08
PTPRO	-5.59		RTP4	2.96
LINC01322	-5.59		MINK1	2.79
KANK1	-5.58		MAFIP	2.79
SLCO4C1	-5.52		LINC0070	2.78
LRAT	-5.48		TONSL-AS	2.70
LYPD6B	-5.47		CST1	2.62
ELMOD1	-5.43		BIK	2.61
AMPH	-5.41		PI16	2.56
FAM184A	-5.41		SLC22A18	2.52
ENPP5	-5.36		NAA11	2.51
PTGFR	-5.34		CEACAM1	2.47
CRISPLD1	-5.26		GCKR	2.44
PTGFRN	-5.26		PRSS36	2.44
MIR137HG	-5.22		RAB40C	2.41
CSRNP3	-5.17		UNC13A	2.36
NDNF	-5.15		OASL	2.35
MAGEA6	-5.13		GPR132	2.31
MIR99AHG	-5.06		GIMAP6	2.30
RERG	-5.05		NAALAD2	2.28
ANGPT1	-4.97		C1QTNF1-	2.27
ZNF300	-4.97		TRIM29	2.25
ARHGDIB	-4.95		NEURL3	2.23

C

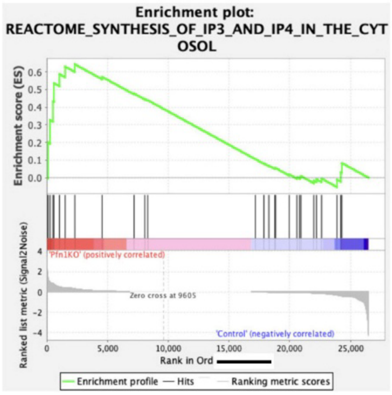

D

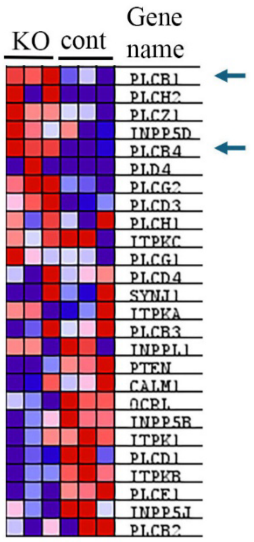

Fig. S3. Summary of transcriptomic findings of control vs Pfn1 KO MDA-231 cells: A) Volcano plot representing differentially expressed genes between control and Pfn1 KO MDA-231 cells (upon Pfn1 KO in MDA-231 cells. **B)** List of top 100 (50 up- and 50-downregulatde) differentially expressed genes in Pfn1 KO relative to control culture. **C-D)** GSEA analyses of transcriptome data (*panel C*) predicts enrichment (normalized enrichment score -1.4; nominal p-value – 0.0) of cytosolic IP_3/IP_4 synthesis-related genes in Pfn1 KO MDA-231 cells; *panel D* shows the associated heat-plot of gene expression (the top 7 listed genes contribute to the enrichment; arrows indicate the PLC isoform genes that are transcriptionally upregulated (~4,0 fold-increase) in a statistically significant manner in Pfn1 KO cells vs their control counterparts.

Journal of Cell Science • Supplementary information
