# Supplementary material for: Molecular insights into profilin 1-dependent regulation of cellular phosphatidylinositol (4,5)-bisphosphate
Source: J Cell Sci. 2026 Jun 12;139(11):jcs265025. doi: 10.1242/jcs.265025 (PMC13286375; doi:10.1242/jcs.265025)
Supplement: Supplementary file 1 [file joces-139-265025_review_history.pdf]

Journal of Cell Science

Review Commons

Molecular insights into profilin 1-dependent regulation of cellular phosphatidylinositol (4,5)-bisphosphate

Andrew Orenberg, Michael Chirumbolo, Ian Eder, Jia-Jun Liu, Silvia Liu, David Gau, Yubo Tang, Klemens Rottner, Jianhua Luo, Gerald V. Hammond and Partha Roy

DOI: 10.1242/jcs.265025

Editor: Michael Way

Review timeline

Submission to Review Commons:	23 December 2025
Submission to Journal of Cell Science:	6 May 2026
Accepted:	8 May 2026

Reviewer 1

Evidence, reproducibility and clarity

Earlier studies have shown that actin-binding protein, profilin, can inhibit the PLC-dependent hydrolysis of PIP2 in vitro and provided evidence that acute profilin-1 (Pfn1) knockdown results in diminished PIP2-levels at the plasma membrane. However, the underlying mechanism by which profilin regulates PIP2-levels in cells has remained elusive. Here, Orenberg at al., show that Pfn1-dependent changes in the plasma membrane PIP2 levels are not transient. Interestingly, they also provide evidence that Pfn1 controls plasma membrane PIP2 levels through its actin-regulating activity and not through directly interacting with PIP2. Finally, they show that loss of Pfn1 also affects the levels of many other lipids in cells.

Majority of the data presented in the manuscript appear of good technical quality, but I have some suggestions to strengthen the manuscript.

1. The quantifications of the PIP2 levels were apparently done simply by measuring the fluorescence intensities of wild-type and knockout cells stained with monoclonal actin-PIP2 antibody. However, the knockout cells appear more spread compared to the wild-type cells (Fig. 1B), and this can possibly affect the quantifications (e.g. there may be more plasma membrane ruffles/folds in the wild-type cells). Thus, I recommend that in all critical quantifications the authors would also use a general plasma membrane marker to confirm that the PIP2-density (and not just morphology of the plasma membrane) is indeed affected by Pfn1-depletion.
2. To get a better idea about which cellular actin filament structures are important for regulating the PIP2-levels at the plasma membrane, one could also use a larger repertoire of actin/myosin inhibitors (CK666, cytochalasin-B, blebbistatin). By using these compounds, one may e.g. uncover if the Arp2/3-nucleated branched actin networks and/or contractile actomyosin structures would specifically contribute to regulation of the plasma membrane PIP2 levels.
3. The effects of PLCb3 silencing on Pfn1-dependent changes in the PIP2 levels are interesting. To gain better insight into the underlying mechanism, one could also check if the levels of active (phosphorylated) PLCb3 are affected upon Pfn1-depletion.
4. In the 'Discussion', the authors speculate that Pfn1 H119E mutant may have more frequent interactions with PIP2 as compared to wild-type Pfn1. This does not make much sense, because Pfn1 binding to PIP2 is very weak (e.g. ref. 28), and it is unlikely that introducing a negatively-charged glutamate would increase its affinity to negatively-charged headgroup of PIP2. Thus, it seems unlikely that Pfn1 would affect the PIP2 content of plasma membrane through direct interactions with PIP2.

© 2026. Published by The Company of Biologists under the terms of the Creative Commons Attribution License (https://creativecommons.org/licenses/by/4.0/).

5. The cell images in Fig. 2A are bit difficult to follow due to the large number of cells in the images. One could perhaps show higher resolution images with few knockout and rescue cells in the same field of view and indicate the rescued cells in these images e.g. with arrows.
6. Please clearly describe in each figure legend what the error bars represent.

Significance

Although this study does not determine the actual mechanism/pathway by which Pfn1 controls plasma membrane PIP2 levels, it nevertheless provides evidence that perturbation of the actin cytoskeleton by loss of actin-binding profilin or with an actin inhibitor latrunculin-B results in decrease in the plasma membrane PIP2, and that PLC-activity is critical for regulation of PIP2 levels downstream of Pfn1 in cells. Therefore, this study presents a valuable contribution to a specific field, and will be interesting to those studying the actin cytoskeleton - plasma membrane interplay.

My expertise: Cytoskeleton research.

Reviewer 2

Evidence, reproducibility and clarity

1. The data show that actin binding-deficient mutants of Pfn1 do not rescue the knockdown. In these experiments, it is critical to quantitate the relative expression levels of the mutants. The model that Pfn1 regulation of PIP2 requires interactions with actin is not really clear - is it due to Pfn1 targeting by actin binding, or Pfn1 regulation of actin itself? Either possibility seems possible, and the experiments do not distinguish them.
2. The knockdown data on PLCbeta is convincing with regard to its role in PIP2 reductions, but the papers does not explain how actin-Pfn1 interactions regulate PLCbeta.
3. The transcriptome data must be provided along with the data in Figure 5 - otherwise it is impossible for the reader to evaluate. The fact that the data is being used in another paper is not an adequate reason for its omission.
4. The PKC substrate data is not convincing. The blots are messy, and there is no quantitation.
5. The calcium data should include statistical analysis of the differences.
6. The discussion of DAG and PA levels is problematic. As the authors are aware, whole cell lipidomics can easily miss small changes in specific compartments. If the authors think that lipid sensor analysis of PM DAG and PA would strengthen the analysis, then this should be included. The large change in PC levels does seem to suggest an alternative source of PA. While the authors present arguments against a role for PLD, this could be directly tested. In any case, the finding of a nearly 100-fold greater change in PC than in PA raises question about what the whole cell PA measurements is really detecting.

Significance

The manuscript by Orenberg et al. is an extension of previous work showing a link between Pfn1 and PM PIP2. While the new data expand the observations, and the PIP2 biosensor data are clean, the proposed model is not really convincing or fully defined - a number of elements are suggestive but not definitive. Several of the data could have multiple explanations (some of which are acknowledged in the discussion). The overriding hypothesis is that Pfn1-actin coupling regulates PLCbeta, but it is not clear how this would happen. Finally, several of the data are not convincing (PKC substrates) or lack statistical analysis (calcium imaging).

Reviewer 3

Evidence, reproducibility and clarity

The manuscript by Orenberg et al. is a well done, well-written paper that provides an in- depth look at the effects of Pfn-1 depletion on PIP2 levels, actin polymerisation and the broader lipidome. I enjoyed reading it, the main conclusions are sound and well-taken & the finding that PIP2 levels correlate with actin polymerization is intriguing as well as the fact that the global

© 2026. Published by The Company of Biologists under the terms of the Creative Commons Attribution License (https://creativecommons.org/licenses/by/4.0/).

lipid to protein ratio changes. This is indicative of the identification of a major player in lipid flux pathways. I have just a few suggestions for control experiments, formulations & figure layout changes that I think will make the paper even better:

- General: Scale bar labels are too small, please also provide time-stamps for time course measurements.
- As with every antibody stain, there is a remaining risk that a change in the cellular context affects an off-target of the antibody (e.g., a protein phosphorylation site). I think that this is not particularly likely, but I'd control for it, which can be done in a straightforward manner: The authors could do a strong-detergent treatment to rule out a potential off-target effect of the antibody (e.g., 0.1% Triton X-100, 1 h). This should remove all (non-amino-) lipids from the sample, including the phosphoinositides. Overall, binding of the antibody should be strongly reduced, fluorescence images should be much dimmer & the effect of the Pfn1 KO should mostly disappear.
- Figure 1: Please show images in a larger zoom, cell details are barely visible (same for Figure 3). I also would not use "PM PIP2 levels" in the legend, as nuclei appear visibly lighter, indicating that some PIP2 is likely present in other membranes. The type of PIP2 staining should be specified in either the Figure itself or in the legend.
- Figure 3: Same comment as for Figure 1, zoomed images would really help, especially for the PM/Cytosol distribution of the PIP2 biosensor.
- The lag time in the dissociation of the PIP2 sensor is interesting, as is the fact that the kinetic of PIP2 biosensor release is (visually) slower. I recommend to do a couple of simple fits to quantify these effects. If my impression holds, this would be a strong support of the author's interpretation that actin depolymerization actually leads to a loss of PM PIP2 - a simple binding/unbinding kinetic would be much closer to the actin depolymerization kinetic.
- Figure 4: Same comment as for Figures 1 and 3, zoomed images would be most helpful
- Figure 5G: It looks like the two conditions were internally normalized. Given that we're looking at differential levels of PIP2/IP3/DAG, I think it is very possible that baseline Ca levels are also different. I'd either report in au or do a global normalization which would also capture any difference between the two conditions. This should also clarify whether there are differences in post-stimulus steady state Ca levels, as it currently looks like.
- Please increase the font size in Figure 6C, this is barely readable

For the discussion:

- Do the authors think that most PIP2 is actually in lipid-protein complexes and actin depolymerization with the corresponding removal of PIP-binding adaptor proteins exposes previously shielded PIP2 molecules to enzymatic hydrolysis?
- The lipidomic changes are extremely interesting. This could indicate a change in overall cellular architecture which goes beyond PIPs. SM/Chol/PC all go down - I'd interpret that this as a relatively lower content of Plasma membrane and ER. It would be interesting to see if the surface to volume ratio of the cell changes - a comparison with total Cardiolipin as a proxy for mitochondrial membrane size could also be informative. It may very well be that the Pfn1 KO effects on structural membrane lipids are the more important finding - but elucidating that mechanism is beyond the scope of the current manuscript. I look forward to learning about it in the next story.

André Nadler

Significance

The manuscript by Orenberg et al. is a well done, well-written paper that provides an in- depth look at the effects of Pfn-1 depletion on PIP2 levels, actin polymerisation and the broader lipidome. I enjoyed reading it, the main conclusions are sound and well-taken & the finding that PIP2 levels correlate with actin polymerization is intriguing as well as the fact that the global lipid to protein ratio changes. This is indicative of the identification of a major player in lipid flux pathways.

© 2026. Published by The Company of Biologists under the terms of the Creative Commons Attribution License (https://creativecommons.org/licenses/by/4.0/).

Manuscript number: RC-2025-03361

Corresponding author(s): Partha, Roy

[Please use this template only if the submitted manuscript should be considered by the affiliate journal as a full revision in response to the points raised by the reviewers.

If you wish to submit a preliminary revision with a revision plan, please use our "Revision Plan" template. ***It is important to use the appropriate template to clearly inform the editors of your intentions.]***

1. General Statements [optional]

We have edited the manuscript with significant amount of new data and textual revisions. We strongly believe that our new DAG/PIP2 biosensor data (requested by reviewer 2) further solidifies our conclusion of actin-binding protein Pfn1 as a negative regulator of PIP2 hydrolysis. This is the first study that reveals 1) Pfn1 regulates PM PIP2 via negatively regulating PLC- mediating PIP2 hydrolysis in a cellular context (and surprisingly in an actin-dependent manner challenging the dogma of a possible role of direct protein-lipid interaction in this context), and 2) Pfn1 is a broad regulator of lipid homeostasis that extends beyond phosphoinositide regulation.

2. Point-by-point description of the revisions

This section is mandatory. Please insert a point-by-point reply describing the revisions that were already carried out and included in the transferred manuscript.

We would like to thank the reviewer for their constructive comments on our manuscript. We have addressed all comments made by the reviewers by additional experimental data, data analyses, and text edits. A detailed point-by-point response to the reviewers is documented below.

Summary of new/amended data panels

Fig 2C (Rev 2): Cell-by-cell quantification of the GFP fluorescence intensity as a surrogate measure of wild-type (WT) vs mutant Pfn1 rescue construct expression levels in B16F1 KO- rescue studies.

Figs 1B, 2A, 3C, 4A, 4C (Rev 1, 3): Inclusion of zoomed images of PIP2 staining of select regions of interests.

Figs 6B, 6D (Rev 2): Quantification of phospho-PKC substrate antibody immunoblots of MDA-231 and B16F1 cells with or without Pfn1 KO.

Fig 3E (*not requested by the reviewers*)**:** Time-lapse images of PIP2 biosensor and F-actin in HEK-293 cells.

Fig 3H (Rev 3): Half-life comparison of LatB-induced PIP2 and F-actin responses

Fig S1 (Rev 1): F-actin and PIP2 staining of MDA-231 cells with or without treatments of myosin inhibitor blebbistatin.

Figs 6G-I (Rev 2, 3): Quantification of various parameters from Ca^{2+} imaging studies.

Fig 6J-M (Rev 2): Images and quantification of correlative PIP2 and DAG biosensor studies in HEK-293 cells.

Fig 7 (*not requested by the reviewers*): A schematic model of how Pfn1 loss leads to PIP2 reduction in cells.

Fig S2 (*not requested by the reviewers*): Effect of Pfn1 knockdown on PI4P in HEK-293 cells. **Fig S3B (Rev 2)**: A list of top 100 (50 up, 50 down) differentially expressed genes in response to Pfn1 KO in MDA-231 cells.

Point-by-Point response

REVIEWER 1:

1. "The quantifications of the PIP2 levels were apparently done simply by measuring the fluorescence intensities of wild-type and knockout cells stained with monoclonal actin-PIP2 antibody. However, the knockout cells appear more spread compared to the wild-type cells (Fig. 1B), and this can possibly affect the quantifications (e.g. there may be more plasma membrane

© 2026. Published by The Company of Biologists under the terms of the Creative Commons Attribution License (https://creativecommons.org/licenses/by/4.0/).

ruffles/folds in the wild-type cells). Thus, I recommend that in all critical quantifications the authors would also use a general plasma membrane marker to confirm that the PIP2-density (and not just morphology of the plasma membrane) is indeed affected by Pfn1-depletion".

Response: For PM PIP2 analysis, we specifically quantified the total rather than the average PM PIP2 staining intensity (as also previously done in other studies – Hammond et al. J. Cell Science 2006; Biochem. J 2009) for three reasons. First, PIP2 is non-uniformly distributed across the PM, and therefore the average intensity calculation collapses a lot of biologically meaningful spatial information. Second, the average intensity calculation is impacted by significant cell shape and area differences that exist between cells within a group as well as between groups. Third, the integrated PM intensity is a better metric of how much total PIP2 is available for metabolic turnover on a cell-by-cell basis. These justifications are now detailed in the revised manuscript.

In our previous study (Ricci et al., J. Biol. Chem 2024, PMID 38141770), we utilized orthogonal techniques (immunostaining, lipid dot blot) in multiple cell lines to demonstrate that total PIP2 as well as PIP2 intensity at the plasma membrane (PM) (based on manual tracing of hundreds of cells in immunostaining experiments) are reduced by silencing Pfn1 expression, and conversely, elevated upon Pfn1 overexpression. We would like to clarify here that in our present study we used an automated pipeline in "cell profiler" to detect cell edges and quantify integrated PM intensity of PIP2 in control vs Pfn1 knockout (KO) cells, and our present findings in Pfn1 KO setting recapitulated our previous findings in transient knockdown setting. While our cell-profile pipeline accurately detects the cell edges, we address the reviewer's comment on confirmation of findings with a PM marker by providing new experimental data in HEK-293 cells transfected with fluorescence biosensors of PIP2 and DAG along with a PM marker (iRFP-Lyn11), which also shows reduction of PIP2 fluorescence staining at the Lyn11-positive PM regions in Pfn1 knockdown cells relative to control cells (**see new data panels Figs 6J, L**).

2. "To get a better idea about which cellular actin filament structures are important for regulating the PIP2-levels at the plasma membrane, one could also use a larger repertoire of actin/myosin inhibitors (CK666, cytochalasin-B, blebbistatin). By using these compounds, one may e.g. uncover if the Arp2/3-nucleated branched actin networks and/or contractile actomyosin structures would specifically contribute to regulation of the plasma membrane PIP2 levels".
Response: We thank the reviewer for this suggestion. We have now evaluated the effect of blebbistatin treatment on PIP2 in MDA-231 cells (now shown **supplementary Fig S1**). A previous study showed that the major effects of blebbistatin on actin cytoskeleton are disintegration of actin stress fibers, softening of cortical actin, and transformation of lamellipodial actin into loose network of accumulated amorphous actin structures that correspond to membrane ruffles (Shutova et al., 2012). These phenotypes were also recapitulated in our experimental settings. In general, blebbistatin-treated cells exhibited protrusive structures in random directions with PIP2 enrichment in peripheral F-actin-rich regions (consistent with the LatB experimental data) and a higher ($p=0.09$) overall cell edge PIP2 staining vs vehicle-treated cells further underscoring the impact of actin cytoskeletal perturbation on PM PIP2.

3. "The effects of PLCβ3 silencing on Pfn1-dependent changes in the PIP2 levels are interesting. To gain better insight into the underlying mechanism, one could also check if the levels of active (phosphorylated) PLCβ3 are affected upon Pfn1-depletion".
Response: We would like to point out that unlike PLCγ, PLCβ is not activated by phosphorylation. While literature has documented that certain site-specific phosphorylations of PLCβ by PKC (in a feedback manner) and PKA, these phosphorylation events, if at all, have inhibitory effect on PLCβ activity. Since our data supports the model that Pfn1 loss leads to an increase in PLC-mediated PIP2 hydrolysis and downstream PKC activation, we feel that probing for such inhibitory feedback phosphorylation events will not provide any mechanistic insights.

4. "In the 'Discussion', the authors speculate that Pfn1 H119E mutant may have more frequent interactions with PIP2 as compared to wild-type Pfn1. This does not make much sense, because Pfn1 binding to PIP2 is very weak (e.g. ref. 28), and it is unlikely that introducing a negativelycharged glutamate would increase its affinity to negatively charged headgroup of PIP2. Thus, it seems unlikely that Pfn1 would affect the PIP2 content of plasma membrane through

© 2026. Published by The Company of Biologists under the terms of the Creative Commons Attribution License (https://creativecommons.org/licenses/by/4.0/).

direct interactions with PIP2".

Response: We did not mean to imply that glutamate substitution of H119 residue would necessarily increase Pfn1's **intrinsic affinity** to negatively charged PIP2. While PIP2 binding of WT vs H119E-Pfn1 has never been quantified in biochemical assays, we previously (Bae et al. PNAS 2010; PMID 21115820) showed that H119E substation does not affect the membrane fraction of ectopically overexpressed Pfn1 in cells. Along this line, Pascal-Goldschmit and colleagues (PMID: 7673143) also showed that analogous mutant H119D-Pfn1 inhibits PLCγ- mediated PIP2 hydrolysis as efficiently as WT-Pfn1, further underscoring the fact that H119D/E- Pfn1 is not defective in membrane phosphoinositide binding. Our data largely supports a model that Pfn1-dependent PIP2 alteration is predominantly related to its actin-regulatory function.

However, since Pfn1's binding to actin and PIP2 are mutually exclusive, we cannot absolutely rule out a minor (possibly insignificant) contribution of Pfn1's ability to block PIP2 hydrolysis by direct PM interaction. We therefore offered a hypothetical scenario where H119E-Pfn1 mutant may have more frequent interaction with PM PIP2 simply because it is not able to interact with actin. We have now better clarified this argument in the "Discussion" section of the revision.

5. "The cell images in Fig. 2A are bit difficult to follow due to the large number of cells in the images. One could perhaps show higher resolution images with few knockout and rescue cells in the same field of view and indicate the rescued cells in these images e.g. with arrows".

Response: As requested by the reviewer, we have now shown zoomed images in **Fig 2A** in the revision.

6. "Please clearly describe in each figure legend what the error bars represent"

Response: We have now clearly mentioned in the Statistics section of "Materials and Methods" that all error bars represent standard deviation unless explicitly mentioned otherwise.

REVIEWER 2

1. "The data show that actin binding-deficient mutants of Pfn1 do not rescue the knockdown. In these experiments, it is critical to quantitate the relative expression levels of the mutants. The model that Pfn1 regulation of PIP2 requires interactions with actin is not really clear - is it due to Pfn1 targeting by actin binding, or Pfn1 regulation of actin itself? Either possibility seems possible, and the experiments do not distinguish them".

Response: We thank the reviewer for these comments. **First**, since GFP and Pfn1 rescue constructs are linked by an IRES, we analyzed GFP fluorescence intensity of cells selected for PIP2 analyses as a surrogate measure for comparing the relative expressions of Pfn1 rescue constructs across the various groups. As per these analyses (based on measurements of hundreds of cells from 3 different experiments), the average GFP expression of cells chosen for PIP2 analyses was found to be comparable between the various Pfn1 KO rescue groups (now shown in **Fig 2C**). Therefore, we argue that our observed phenotypic differences related to PIP2 are not confounded by the expressions of various Pfn1 rescue constructs.

Second, it is known that Pfn1 loss leads to pronounced reduction in lamellipodial F-actin content (as shown in **Figs 3A-B**). Our LatB experimental data (**Figs 3E-G**) show that actin depolymerization leads to pronounced PM PIP2 reduction within minutes. Based on these findings, taken together additional evidence for increased basal PLC activity signature readouts in Pfn1-deficient cells (i.e. greater baseline PKC activity, greater PM DAG/PIP2 ratio from biosensor studies as recommended by the reviewer (**new data** – shown in **Figs 6J-M**)), we postulate (*concurring with **Reviewer 3***) that disruption of cortical cytoskeleton (possibly also accompanied by removal of PIP2-binding adaptor proteins) may enhance PIP2's accessibility to hydrolytic enzymes. In fact, two previous studies (*Cho et al., PNAS, 2005 and Andrade et al., Scientific Reports 2015*) have demonstrated that actin filament disruption increases PM mobility of PIP2. There is also evidence for actin depolymerization-induced uncaging of PLC from the cortical actin network (*Huang et al, Planta, 2009*). Therefore, in principle, Pfn1 loss may cause more frequent PLC-PIP2 interaction and enhance baseline PIP2 hydrolysis by either increasing PM diffusion of PIP2 and/or uncaging of PLC. We have now included a schematic working model (**Fig 7**) to illustrate this concept and added these points in the discussion. However, a direct demonstration of increased PIP2 accessibility

© 2026. Published by The Company of Biologists under the terms of the Creative Commons Attribution License (https://creativecommons.org/licenses/by/4.0/).

of PLC in Pfn1-deficient cells is beyond the scope of the present – this is something we will pursue in the future.

2. “The knockdown data on PLCbeta is convincing with regard to its role in PIP2 reductions, but the papers does not explain how actin-Pfn1 interactions regulate PLCbeta”.
Response: Please see our detailed response to the previous comment that specifically addresses how we envision Pfn1 negatively regulates PLC-mediated PIP2 hydrolysis via modulating actin cytoskeleton.

3. “The transcriptome data must be provided along with the data in Figure 5 - otherwise it is impossible for the reader to evaluate. The fact that the data is being used in another paper is not an adequate reason for its omission”.
Response: The transcriptomic data is now displayed in Supplementary **Figure S3**, where we have now listed top 100 (50 up, 50 down) differentially expressed genes in response to Pfn1 KO in MDA-231 cells (see *panel B* in Fig S2). We are in the process of submitting the FASTA file to GEO database.

4. “The PKC substrate data is not convincing. The blots are messy, and there is no quantitation”
Response: Since phospho-PKC substrate antibody is supposed to recognize all phosphorylated proteins by PKC, we expect to see multiple bands. The intensity of each lane in entirety is approximative of PKC activity by detecting proteins at multiple molecular weights phosphorylated at their serine residues. We have replaced the B16 generated data with a better-quality blot and added quantifications with statistical analysis (**Figs 6B, D**).

5. “The calcium data should include statistical analysis of the differences”.
Response: We have now performed statistical analyses of the calcium data. Specifically, we compared the peak amplitude, integrated Ca^{2+} signal (area under the curve), and the post-stimulation resting value between control and Pfn1 knockdown groups. As per these analyses, we did not see any significant difference in either the peak amplitude or integrated Ca^{2+} signal between the control and Pfn1 knockdown groups, further underscoring the fact that Pfn1 loss does not necessarily confer cells an increased ability to respond to agonists (i.e. LPA-induced GPCR activation in this specific case). However, we noted that the post-stimulation resting Ca^{2+} signal was elevated in Pfn1-deficient cells relative to control cells ($p<0.01$), a feature that could result from increased basal PIP2 hydrolysis and/or reduced re-uptake of cytosolic Ca^{2+} by endoplasmic reticulum and/or reduced efficiency of Ca^{2+} export. These analyses are now included in **Figs 6G-I** in the revision.

6. “The discussion of DAG and PA levels is problematic. As the authors are aware, whole cell lipidomics can easily miss small changes in specific compartments. If the authors think that lipid sensor analysis of PM DAG and PA would strengthen the analysis, then this should be included. The large change in PC levels does seem to suggest an alternative source of PA. While the authors present arguments against a role for PLD, this could be directly tested. In any case, the finding of a nearly 100-fold greater change in PC than in PA raises question about what the whole cell PA measurements is really detecting”.
Response: We thank the reviewer for these comments and experimental suggestions. **First**, we completely agree with the reviewer that whole cell lipidomic analyses fail to detect small changes in specific compartment; we mention this point in the revision. In the revision, we have displayed our lipids of interest as individual line plots connecting control and Pfn1 KO group experiment-by-experiment to show the trend of lipid change in each experiment. As per these analyses, in 4 out 5 experiments, the total DAG increased in Pfn1 KO cells. However, the large experiment-to-experiment variability in the absolute content as well as Pfn1-dependent changes in DAG precluded us from achieving statistical significance between the two groups. The large variability in the measured DAG content in our experiments is not totally surprising since cellular DAG level is known to fluctuate with growth and/or impacted by unintended changes in the chemical parameters of culture condition. However, the largest pool of DAG is in ER/golgi, and since whole cell lipidomic measurements fail to reveal PM DAG due to PIP2 hydrolysis, as per reviewer’s

© 2026. Published by The Company of Biologists under the terms of the Creative Commons Attribution License (https://creativecommons.org/licenses/by/4.0/).

recommendation, we now include lipid biosensor experimental data (**Fig 6J-M**) of control vs Pfn1 knockdown HEK-293 cells to demonstrate that PM DAG-to-PIP2 ratio (an indicator of the basal PIP2 hydrolysis efficiency) is increased upon Pfn1 depletion. We believe that these new correlative PIP2/DAG biosensor data further strengthen our conclusion.

Regarding the reviewer's comment on the orders of change in PC vs PA, we clearly mentioned in the original discussion that it is highly unlikely that PA increase in Pfn1-deficient cells is reflective of increased PLD-mediated conversion of PC for two reasons. First, we saw disproportionate orders of magnitude of changes in the content of PA (~3000 pmol/mg increase) vs PC (>200,000 pmol/mg decrease) in response to Pfn1 KO in MDA-231 cells. Second and more importantly, since monomeric actin directly binds to and inhibits the activity of PLD, the expected increased G-to-F-actin ratio in Pfn1-deficient cells, if at all, would likely result in diminished PLD activity reducing PLD-mediated conversion of PC to PA.

In our opinion, since DAG is the direct hydrolysis product of PIP2 and we are now able to demonstrate elevated PM DAG-to-PIP2 ratio in Pfn1-deficient cells in biosensor experiments, PA biosensor studies are not necessary.

REVIEWER #3

1. "General: Scale bar labels are too small, please also provide time-stamps for time course measurements"

Response: These concerns have been addressed in the revision.

2. "As with every antibody stain, there is a remaining risk that a change in the cellular context affects an off-target of the antibody (e.g., a protein phosphorylation site). I think that this is not particularly likely, but I'd control for it, which can be done in a straightforward manner: The authors could do a strong-detergent treatment to rule out a potential off-target effect of the antibody (e.g., 0.1% Triton X-100, 1 h). This should remove all (non-amino-) lipids from the sample, including the phosphoinositides. Overall, binding of the antibody should be strongly reduced, fluorescence images should be much dimmer & the effect of the Pfn1 KO should mostly disappear."

Response: The PIP2 antibody used in the present study is a well-vetted and widely used antibody in literature. Notably, two papers published by Dr. Hammond (one of the co-authors), an expert in phosphoinositide signaling, previously showed selectivity of this antibody by blocking with lipids, neomycin, and PH-domain of PIP2-binding proteins (Hammond et al, J. Cell Sci, 2006; Biochem J. 2009). We cite these papers in the revision.

3. "Figure 1: Please show images in a larger zoom, cell details are barely visible (same for Figure 3). I also would not use "PM PIP2 levels" in the legend, as nuclei appear visibly lighter, indicating that some PIP2 is likely present in other membranes. The type of PIP2 staining should be specified in either the Figure itself or in the legend."

Response: We would like to clarify here that we used an automated pipeline in "cell profiler" to detect cell edges and quantify integrated PM intensity of PIP2 in control vs Pfn1 knockout (KO) cells; so nuclear membrane PM is not accounted for in the analyses. We have zoomed PIP2 images in Figure 1 as the reviewer suggested. These changes are incorporated in the revision.

4. "Figure 3: Same comment as for Figure 1, zoomed images would really help, especially for the PM/Cytosol distribution of the PIP2 biosensor"

Response: Zoomed images of Fig 3 have been provided in the revision.

5. "The lag time in the dissociation of the PIP2 sensor is interesting, as is the fact that the kinetic of PIP2 biosensor release is (visually) slower. I recommend to do a couple of simple fits to quantify these effects. If my impression holds, this would be a strong support of the author's interpretation that actin depolymerization actually leads to a loss of PM PIP2 - a simple binding/unbinding kinetic would be much closer to the actin depolymerization kinetic".

Response: As suggested by the reviewer, we have done curve fitting of these data to calculate the half-life of F-actin and PIP2 (results shown in **Fig 3H**). As per these calculations, the mean half-

© 2026. Published by The Company of Biologists under the terms of the Creative Commons Attribution License (https://creativecommons.org/licenses/by/4.0/).

life of PIP2 (~ 1min) is significantly longer than that of F-actin (~2.2 min) which further supports our interpretation that actin depolymerization leads to a loss of PM PIP2.

6. “Figure 4: Same comment as for Figures 1 and 3, zoomed images would be most helpful.”
Response: Zoomed images have been provided in the revision.

7. “Figure 5G: It looks like the two conditions were internally normalized. Given that we're looking at differential levels of PIP2/IP3/DAG, I think it is very possible that baseline Ca levels are also different. I'd either report in au or do a global normalization which would also capture any difference between the two conditions. This should also clarify whether there are differences in post-stimulus steady state Ca levels, as it currently looks like”.
Response: Since we used a transfectable Ca2+ biosensor (GCaMP), to account for cell-to-cell variation in the actual expression of the biosensor, we had to baseline-corrected GCaMP fluorescence by normalizing each kinetic datapoint readout to the average pre-stimulation value on a cell-by-cell basis. However, we have now performed additional analyses. Specifically, we calculated the peak amplitude, integrated Ca^{2+} signal (area under the curve), and the post-stimulation resting value for each of the two groups. As per these analyses, we did not see any significant difference in either the peak amplitude or integrated Ca^{2+} signal between the control and Pfn1 knockdown groups, further underscoring the fact that Pfn1 loss does not necessarily confer cells an increased ability to respond to agonists (i.e. LPA-induced GPCR activation in this specific case). However, we noted that the post-stimulation resting Ca^{2+} signal was elevated in Pfn1-deficient cells relative to control cells ($p<0.01$), a feature that could result from increased basal PIP2 hydrolysis and/or reduced re-uptake of cytosolic Ca^{2+} by endoplasmic reticulum and/or reduced efficiency of Ca^{2+} export. These analyses are now included in **Figs 6G-I** in the revision.

8. “Please increase the font size in Figure 6C, this is barely readable”.
Response: We have now replaced that panel with one with bigger font texts.

9. “Do the authors think that most PIP2 is actually in lipid-protein complexes and actin depolymerization with the corresponding removal of PIP-binding adaptor proteins exposes previously shielded PIP2 molecules to enzymatic hydrolysis?”
Response: Yes, we certainly think that is the most likely scenario. Please see our detailed response to Reviewer 2’s comment #1. We have now clearly included this in the discussion and added a schematic mechanistic model to better illustrate our thinking (**Figure 7**).

10. “The lipidomic changes are extremely interesting. This could indicate a change in overall cellular architecture which goes beyond PIPs. SM/Chol/PC all go down - I'd interpret that this as a relatively lower content of Plasma membrane and ER. It would be interesting to see if the surface to volume ratio of the cell changes - a comparison with total Cardiolipin as a proxy for mitochondrial membrane size could also be informative. It may very well be that the Pfn1 KO effects on structural membrane lipids are the more important finding - but elucidating that mechanism is beyond the scope of the current manuscript. I look forward to learning about it in the next story”.
Response: We thank the reviewer for this insightful comment. However, this is something we would consider as a scope of future studies.

Original submission

First decision letter

MS ID#: jcs.265025

MS Title: Molecular insights into Profilin1-dependent regulation of cellular phosphatidylinositol-(4,5)-bisphosphate

© 2026. Published by The Company of Biologists under the terms of the Creative Commons Attribution License (https://creativecommons.org/licenses/by/4.0/).

Authors: Andrew Orenberg; Michael Chirumbolo; Ian Eder; Jia-Jun Liu; Silvia Liu; David Gau; Yubo Tang; Klemens Rottner; Jianhua Luo; Gerald Hammond; Partha Roy

Article Type: Review Commons Transfer

Dear Dr Roy,

Based on your revisions to the reviewers questions I am happy to tell you that your manuscript has been accepted for publication in Journal of Cell Science, pending standard publication integrity checks.

Thank you for sending your manuscript to Journal of Cell Science through Review Commons.

© 2026. Published by The Company of Biologists under the terms of the Creative Commons Attribution License (https://creativecommons.org/licenses/by/4.0/).
